# Hybrid cloud and cluster computing paradigms for life science applications

**DOI:** 10.1186/1471-2105-11-S12-S3

**Published:** 2010-12-21

**Authors:** Judy Qiu, Jaliya Ekanayake, Thilina Gunarathne, Jong Youl Choi, Seung-Hee Bae, Hui Li, Bingjing Zhang, Tak-Lon Wu, Yang Ruan, Saliya Ekanayake, Adam Hughes, Geoffrey Fox

**Affiliations:** 1 School of Informatics and Computing, Indiana University, Bloomington, IN 47405, USA; 2 Pervasive Technology Institute, Indiana University, Bloomington, IN 47408, USA

## Abstract

**Background:**

Clouds and MapReduce have shown themselves to be a broadly useful approach to scientific computing especially for parallel data intensive applications. However they have limited applicability to some areas such as data mining because MapReduce has poor performance on problems with an iterative structure present in the linear algebra that underlies much data analysis. Such problems can be run efficiently on clusters using MPI leading to a hybrid cloud and cluster environment. This motivates the design and implementation of an open source Iterative MapReduce system Twister.

**Results:**

Comparisons of Amazon, Azure, and traditional Linux and Windows environments on common applications have shown encouraging performance and usability comparisons in several important non iterative cases. These are linked to MPI applications for final stages of the data analysis. Further we have released the open source Twister Iterative MapReduce and benchmarked it against basic MapReduce (Hadoop) and MPI in information retrieval and life sciences applications.

**Conclusions:**

The hybrid cloud (MapReduce) and cluster (MPI) approach offers an attractive production environment while Twister promises a uniform programming environment for many Life Sciences applications.

**Methods:**

We used commercial clouds Amazon and Azure and the NSF resource FutureGrid to perform detailed comparisons and evaluations of different approaches to data intensive computing. Several applications were developed in MPI, MapReduce and Twister in these different environments.

## Background

Cloud computing [1] is at the peak of the Gartner technology hype curve [2], but there are good reasons to believe that it is for real and will be important for large scale scientific computing:

1) Clouds are the largest scale computer centers constructed, and so they have the capacity to be important to large-scale science problems as well as those at small scale.

2) Clouds exploit the economies of this scale and so can be expected to be a cost effective approach to computing. Their architecture explicitly addresses the important fault tolerance issue.

3) Clouds are commercially supported and so one can expect reasonably robust software without the sustainability difficulties seen from the academic software systems critical to much current cyberinfrastructure.

4) There are 3 major vendors of clouds (Amazon, Google, and Microsoft) and many other infrastructure and software cloud technology vendors including Eucalyptus Systems, which spun off from UC Santa Barbara HPC research. This competition should ensure that clouds develop in a healthy, innovative fashion. Further attention is already being given to cloud standards [3].

5) There are many cloud research efforts, conferences, and other activities including Nimbus [4], OpenNebula [5], Sector/Sphere [6], and Eucalyptus [7].

6) There are a growing number of academic and science cloud systems supporting users through NSF Programs for Google/IBM and Microsoft Azure systems. In NSF OCI, FutureGrid [8] offers a cloud testbed, and Magellan [9] is a major DoE experimental cloud system. The EU framework 7 project VENUS-C [10] is just starting with an emphasis on Azure.

7) Clouds offer attractive "on-demand" elastic and interactive computing.

Much scientific computing can be performed on clouds [11], but there are some well-documented problems with using clouds, including:

1) The centralized computing model for clouds runs counter to the principle of "bringing the computing to the data", and bringing the "data to a commercial cloud facility" may be slow and expensive.

2) There are many security, legal, and privacy issues [12] that often mimic those of the Internet which are especially problematic in areas such health informatics.

3) The virtualized networking currently used in the virtual machines (VM) in today’s commercial clouds and jitter from complex operating system functions increases synchronization/communication costs. This is especially serious in large-scale parallel computing and leads to significant overheads in many MPI applications [13-15]. Indeed, the usual (and attractive) fault tolerance model for clouds runs counter to the tight synchronization needed in most MPI applications. Specialized VMs and operating systems can give excellent MPI performance [16] but we will consider commodity approaches here. Amazon has just announced Cluster Compute instances in this area.

4) Private clouds do not currently offer the rich platform features seen on commercial clouds [17].

Some of these issues can be addressed with customized (private) clouds and enhanced bandwidth from research systems like TeraGrid to commercial cloud networks. However it seems likely that clouds will not supplant traditional approaches for very large-scale parallel (MPI) jobs in the near future. Thus we consider a hybrid model with jobs running on classic HPC systems, clouds, or both as workflows could link HPC and cloud systems. Commercial clouds support "massively parallel" or “many tasks” applications, but only those that are loosely coupled and so insensitive to higher synchronization costs. We focus on the MapReduce programming model [18], which can be implemented on any cluster using the open source Hadoop [19] software for Linux or the Microsoft Dryad system [20,21] for Windows. MapReduce is currently available on Amazon systems, and we have developed a prototype MapReduce for Azure.

### Results

#### Metagenomics - a data intensive application vignette

**Figure 1:**
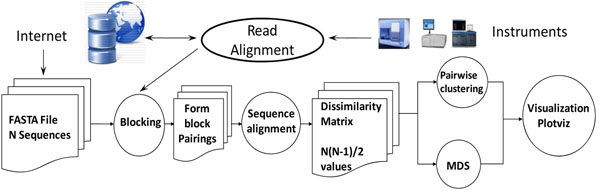
Pipeline for analysis of metagenomics Data

**Figure 2:**
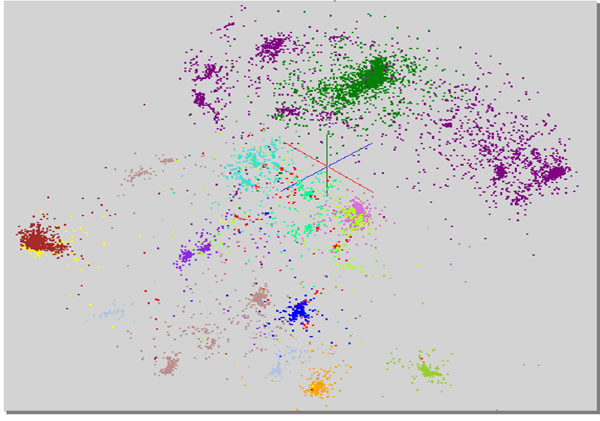
Results of 17 clusters for full sample using Sammon’s version of MDS for visualization [24]

The study of microbial genomes is complicated by the fact that only small number of species can be isolated successfully and the current way forward is metagenomic studies of culture-independent, collective sets of genomes in their natural environments. This requires identification of as many as millions of genes and thousands of species from individual samples. New sequencing technology can provide the required data samples with a throughput of 1 trillion base pairs per day and this rate will increase. A typical observation and data pipeline [22] is shown in Figure 1 with sequencers producing DNA samples that are assembled and subject to further analysis including BLAST-like comparison with existing datasets as well as clustering and visualization to identify new gene families. Figure 2 shows initial results from analysis of 30,000 sequences with clusters identified and visualized using dimension reduction to map to three dimensions with Multi-dimensional scaling MDS [23]. The initial parts of the pipeline fit the MapReduce or many-task Cloud model but the latter stages involve parallel linear algebra.

**Figure 3:**
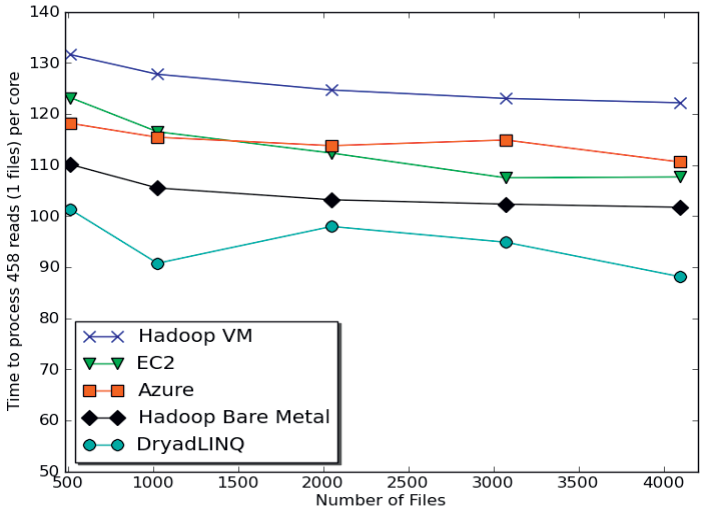
Time to process a single biology sequence file (458 reads) per core with different frameworks[24]

State of the art MDS and clustering algorithms scale like O(N^2^) for N sequences; the total runtime for MDS and clustering is about 2 hours each on a 768 core commodity cluster obtaining a speedup of about 500 using a hybrid MPI-threading implementation on 24 core nodes. The initial steps can be run on clouds and include the calculation of a distance matrix of N(N-1)/2 independent elements. Million sequence problems of this type will challenge the largest clouds and the largest TeraGrid resources. Figure 3 looks at a related sequence assembly problem and compares performance of MapReduce (Hadoop, DryadLINQ) with and without virtual machines and the basic Amazon and Microsoft clouds. The execution times are similar (range is 30%) showing that this class of algorithm can be effectively run on many different infrastructures and it makes sense to consider the intrinsic advantages of clouds described above. In recent work we have looked hierarchical methods to reduce O(N^2^ ) execution time to O(NlogN) or O(N) and allow loosely-coupled cloud implementation with initial results on interpolation methods presented in [23].

One can study in [22,25,26] which applications run well on MapReduce and relate this to an old classification of Fox [27]. One finds that Pleasingly Parallel and a subset of what was called “Loosely Synchronous” applications run on MapReduce. However, current MapReduce addresses problems with only a single (or a “few”) MapReduce iterations, whereas there are a large set of data parallel applications that involve many iterations and are not suitable for basic MapReduce. Such iterative algorithms include linear algebra and many data mining algorithms [28], and here we introduce the open source Twister to address these problems. Twister [25,29] supports applications needing either a few iterations or many iterations using a subset of MPI - reduction and broadcast operations and not the latency sensitive MPI point-to-point operations.

Twister [29] supports iterative computations of the type needed in clustering and MDS [23]. This programming paradigm is attractive as Twister supports all phases of the pipeline in Figure 1 with performance that is better or comparable to the basic MapReduce and on large enough problems similar to MPI for the iterative cases where basic MapReduce is inadequate. The current Twister system is just a prototype and further research will focus on scalability and fault tolerance. The key idea is to combine the fault tolerance and flexibility of MapReduce with the performance of MPI.

**Figure 4:**
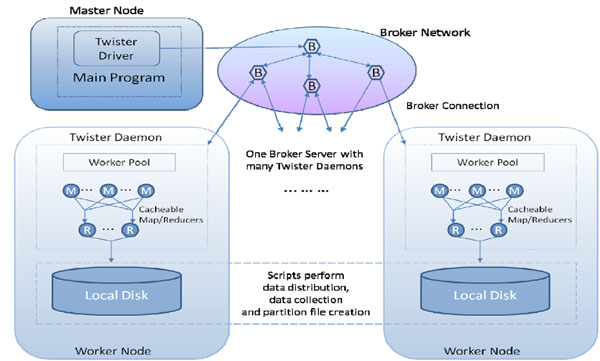
Current Twister Prototype

**Figure 5:**
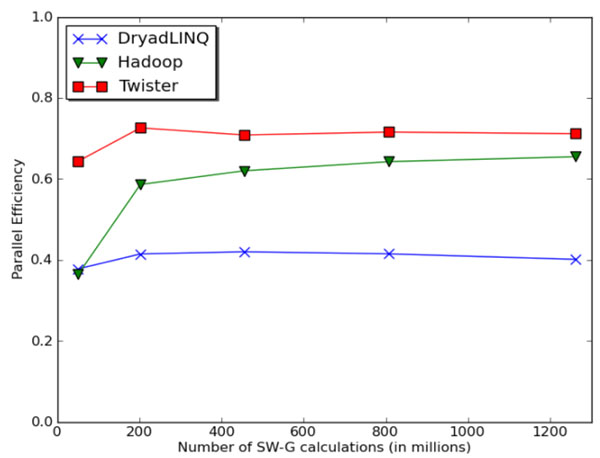
Parallel Efficiency of the different parallel runtimes for the Smith Waterman Gotoh algorithm for distance computation

**Figure 6:**
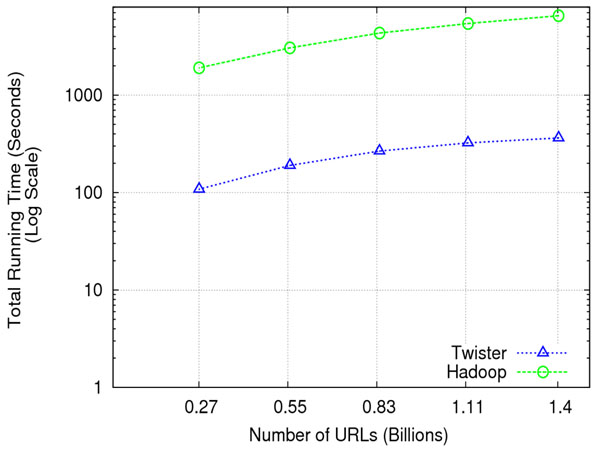
Total running time for 20 iterations of PageRank algorithm on ClueWeb data with Twister and Hadoop on 256 cores

The current Twister, shown in Figure 4, is a distributed in-memory MapReduce runtime optimized for iterative MapReduce computations. It reads data from local disks of the worker nodes and handles the intermediate data in distributed memory of the worker nodes. All communication and data transfers are handled via a Publish/Subscribe messaging infrastructure. Twister comprises three main entities: (i) Twister Driver or Client that drives the entire MapReduce computation, (ii) Twister Daemon running on every worker node, and (iii) the broker network. We present two representative results of our initial analysis of Twister [25,29] in Figure 5 and 6.

We showed “doubly data parallel” (all pairs) application like pairwise distance calculation using Smith Waterman Gotoh algorithm can be implemented with Hadoop, Dyrad, and MPI [30]. Further, Figure 5 shows a classic MapReduce application already studied in Figure 2 and demonstrates that Twister will perform well in this limit, although its iterative extensions are not needed. We use the conventional efficiency defined as T(1)/(pT(p)), where T(p) is runtime on p cores. The results shown in Figure 5 were obtained using 744 cores (31 24-core nodes). Twister outperforms Hadoop because of its faster data communication mechanism and the lower overhead in the static task scheduling. Moreover, in Hadoop each map/reduce task is executed as a separate process, whereas Twister uses a hybrid approach in which the map/reduce tasks assigned to a given daemon are executed within one Java Virtual Machine (JVM). The lower efficiency in DryadLINQ shown in Figure 5 was mainly due to an inefficient task scheduling mechanism used in the initial academic release [21]. We also investigated Twister PageRank performance using a ClueWeb data set [31] collected in January 2009. We built the adjacency matrix using this data set and tested the page rank application using 32 8-core nodes. Figure 6 shows that Twister performs much better than Hadoop on this algorithm [32], which has the iterative structure, for which Twister was designed.

## Conclusions

We have shown that MapReduce gives good performance for several applications and is comparable in performance to but easier to use [33] (from its high level support of parallelism) than conventional master-worker approaches, which are automated in Azure with its concept of roles. However many data mining steps cannot efficiently use MapReduce and we propose a hybrid cloud-cluster architecture to link MPI and MapReduce components. We introduced the MapReduce extension Twister [25,29] to allow a uniform programming paradigm across all processing steps in a pipeline typified by Figure 1.

## Methods

We used three major computational infrastructures: Azure, Amazon and FutureGrid. FutureGrid offers a flexible environment for our rigorous benchmarking of virtual machine and "bare-metal" (non-VM) based approaches, and an early prototype of FutureGrid software was used in our initial work. We used four distinct parallel computing paradigms: the master-worker model, MPI, MapReduce and Twister.

## List of abbreviations

MPI: Message Passing Interface; NSF: National Science Fundation; UC Santa Barbara HPC Research: University of California Santa Barbara High Performance Computing Research; OCI: Office of Cyberinfrastructure; DOE: Department of Energy; EU: European Union; VM: Virtual Machine; HPC: High Performance Computing; DNA: Deoxyribonucleic Acid; BLAST: Basic Local Alignment Search Tool; MDS: Multidimensional Scaling; JVM: Java Virtual Machine

## Competing interests

The authors declare that they have no competing interests.

## Authors' contributions

JQ participated in study of the hybrid Cloud and clustering computing pipeline model. JE, TG, JQ, HL, BZ, TLW carried out Smith Waterman Gotoh sequence alignment using Twister, DryadLINQ, Hadoop, and MPI. JYC, SHB and JE, contributed on parallel MDS and GTM using MPI and Twister. YR, ES and AH studied workflow and job scheduling on clusters. GF participated in study of Cloud and parallel computing research issues.
